# Genome-wide identification and gene-editing of pigment transporter genes in the swallowtail butterfly *Papilio xuthus*

**DOI:** 10.1186/s12864-021-07400-z

**Published:** 2021-02-17

**Authors:** Guichun Liu, Wei Liu, Ruoping Zhao, Jinwu He, Zhiwei Dong, Lei Chen, Wenting Wan, Zhou Chang, Wen Wang, Xueyan Li

**Affiliations:** 1 School of Ecology and Environment, Northwestern Polytechnical University, Xi’an, 710072 Shanxi China; 2 State Key Laboratory of Genetic Resources and Evolution, Kunming Institute of Zoology, Chinese Academy of Sciences, Kunming, 650223 Yunnan China; 3 Kunming College of Life Science, University of Chinese Academy of Sciences, Kunming, 650204 China; 4 Center for Excellence in Animal Evolution and Genetics, Kunming, 650223 Yunnan China

**Keywords:** ATP-binding cassette (ABC) transporters, Rab transporters, *Papilio xuthus*, CRISPR/Cas9, Transcriptome

## Abstract

**Background:**

Insect body coloration often functions as camouflage to survive from predators or mate selection. Transportation of pigment precursors or related metabolites from cytoplasm to subcellular pigment granules is one of the key steps in insect pigmentation and usually executed via such transporter proteins as the ATP-binding cassette (ABC) transmembrane transporters and small G-proteins (e.g. Rab protein). However, little is known about the copy numbers of pigment transporter genes in the butterfly genomes and about the roles of pigment transporters in the development of swallowtail butterflies.

**Results:**

Here, we have identified 56 ABC transporters and 58 Rab members in the genome of swallowtail butterfly *Papilio xuthus*. This is the first case of genome-wide gene copy number identification of ABC transporters in swallowtail butterflies and Rab family in lepidopteran insects. Aiming to investigate the contribution of the five genes which are orthologous to well-studied pigment transporters (ABCG: *white*, *scarlet*, *brown* and *ok*; Rab: *lightoid*) of fruit fly or silkworm during the development of swallowtail butterflies, we performed CRISPR/Cas9 gene-editing of these genes using *P. xuthus* as a model and sequenced the transcriptomes of their morphological mutants. Our results indicate that the disruption of each gene produced mutated phenotypes in the colors of larvae (cuticle, testis) and/or adult eyes in G0 individuals but have no effect on wing color. The transcriptomic data demonstrated that mutations induced by CRISPR/Cas9 can lead to the accumulation of abnormal transcripts and the decrease or dosage compensation of normal transcripts at gene expression level. Comparative transcriptomes revealed 606 ~ 772 differentially expressed genes (DEGs) in the mutants of four ABCG transporters and 1443 DEGs in the mutants of *lightoid*. GO and KEGG enrichment analysis showed that DEGs in ABCG transporter mutants enriched to the oxidoreductase activity, heme binding, iron ion binding process possibly related to the color display, and DEGs in *lightoid* mutants are enriched in glycoprotein binding and protein kinases.

**Conclusions:**

Our data indicated these transporter proteins play an important role in body color of *P. xuthus*. Our study provides new insights into the function of ABC transporters and small G-proteins in the morphological development of butterflies.

**Supplementary Information:**

The online version contains supplementary material available at 10.1186/s12864-021-07400-z.

## Background

Butterflies display a diversity of body color among and within species in their different development stages, especially larvae and adults, serving diverse and crucial functions in sexual selection, predator avoidance, and thermoregulation [1]. Like other insects, the metabolites from three main pigmentation pathways (i.e., tyrosine-derived melanin, tryptophan-derived ommochromes and guanine-derived pteridines) and other related metabolites (i.e., uric acid etc.) mainly contribute to color pattern in butterflies [2, 3]. Tyrosine-derived melanin metabolites are well known to play central roles in body color of all kinds of insects [4]. Tryptophan-derived ommochromes and guanine-derived pteridine have been verified to contribute to eye color in many insects independently (e.g., flour beetle *Tribolium casstaneum*) [5–9], or jointly (e.g., fruit fly *Drosophila melanogaster*, cotton ballworm *Helicoverpa armigera*, water strider *Limnogonus franciscanus*) [10–14]; they also play important roles in coloration of larval epidermis and wing etc. [15, 16]. In addition, the fourth pigment, i.e., papiliochrome, is unique to swallowtail butterflies (Papilionidae) and biosynthesized from one tyrosine-derived metabolite (N-β-alanyldopamine) and one tryptophan-derived metabolite (kynurenine) [17, 18]. In insects, pigments are biosynthesized in epidermal cells through a development process that includes pigment patterning and synthesis [18]. During the process, one of the key steps is the transportation of pigment precursors or related metabolites, which are usually executed via such transporter such as ATP-binding cassette (ABC) proteins, Rab proteins etc. [12, 19].

ABC family is one of the largest transporter families and present in all living organisms [20, 21]. They can be classified into seven subfamilies in human [22, 23] or eight subfamilies (A-H) in arthropods [24]. The majority of these ABC proteins function as primary-active transporters. For ABC transporters, ATP binding and hydrolyzing in the nucleotide-binding domains (NBDs) is a necessary process to transport a wide spectrum of substrates (e.g., amino acids, sugars, heavy metal ions and conjugates, peptides, lipids, polysaccharides, xenobiotic and chemotherapeutic drugs) via the integral transmembrane domains (TMDs) across lipid membranes [24, 25]. Notably, ABCG subfamily includes such well-studied ABC members as *white*, *scarlet* and *brown* in *D. melanogaster*, which are involved in the uptake of pigment precursors in ommochromes and pteridines pathways in the development of cells of Malpighian tubules and compound eyes [26–29]. The functional experiments from such a few non-dipteran insects as Lepidoptera (including a few moths and one nymphid African butterfly *Bicyclus anynana*), Coleoptera, Hemiptera, Orthoptera also confirmed the important roles of these ABCG members (especially *white* and *scarlet*) in pigmentation [8–10, 14, 30–35]. It is very interesting that no morphologically mutated phenotypes were observed in *H. armigera* of Lepidoptera after the *brown* gene was disrupted [10]. Nevertheless, another ABCG gene, *ok,* a paralog of *brown*, was identified in Lepidoptera (*B. mori*, *H. armigera*) and verified to play an important role in the development of larval epidermis or/and adult eyes [3, 10]. Another kind of notable transporter proteins are Rab proteins, which are small (21–25 kDa) monomeric GTPase/GTP-binding proteins and found in organisms ranging from yeast to humans with different gene copies [19]. They are known to be involved in intracellular vesicle transport [36]. Among 33 Rab genes identified in the genome of *D. melanogaster*, Rab32/RP1, encoded by gene *lightoid*, plays an important role in eye color via participating in biogenesis or degradation of pigment granules [37–39]. However, nothing is known for function of *lightoid* in other insects except for fruit fly and silkworm.

The experiments from *Drosophila* and other insects demonstrate the important roles of such transporter proteins as ABCG members and Rab proteins in pigmentations [12, 18, 19]. However, it is still not sure whether these findings hold for swallowtail butterflies (Papilionidae), the most historically significant group of butterflies (Papilionoidea) because of their phylogenetic basal position to all other butterflies and their morphological diversity. Moreover, it is not known how these transporter genes affect the expression profiling of other related genes. In addition, we aim to test if these transporters contribute to the biosynthesis of papiliochrome in swallowtail butterflies by transporting the precursor (kynurenine) of tryptophan-derived metabolites, as postulated in our previous work [2]. The swallowtail butterfly *P. xuthus* is an intriguing species commonly used in butterfly research because of both their enigmatically morphological changes in ontogeny and their well-studied biology as well as ease of breeding [2, 40–42]. Here, we systematically identified potential ABC transporters and Rab protein family in the genome of *P. xuthus*. Then, we investigate the contributions of five of them, which are orthologous to well-studied pigment transporters (ABCG: *white*, *scarlet*, *brown* and *ok*; Rab: *lightoid*) in fruit fly, in the development of *P. xuthus* via CRISPR/Cas9 technology which is widely used in insect [43]. Combining comparative transcriptomics of mutants and wild-types, we provide new insights into the function of ABC transporters and small G-proteins in the morphological development of swallowtail butterflies.

## Results

### Identification and phylogenetic analysis of ABC and Rab transporters in *P. xuthus*

**Fig. 1:**
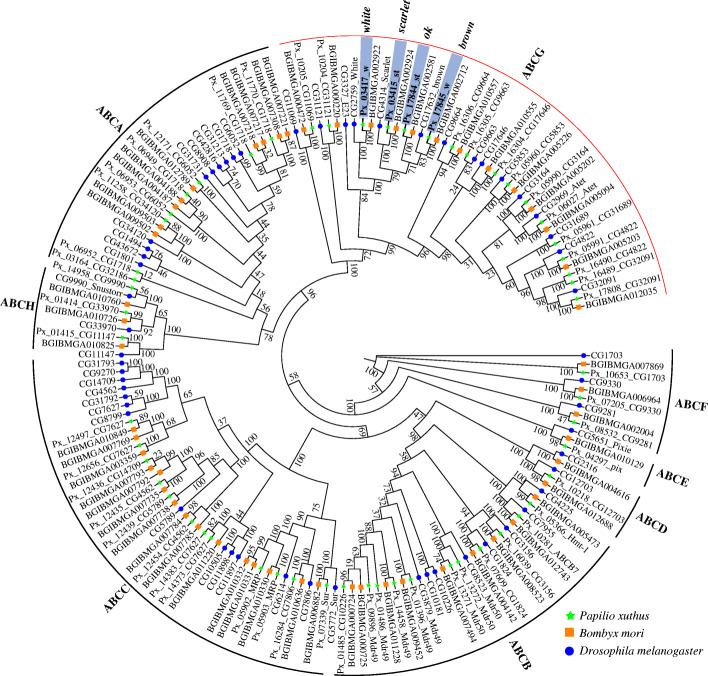
Phylogenetic tree of ATP-binding cassette (ABC) transporters of *Papilio xuthus* (Px), *Bombyx mori* (BGIBM) and *Drosophila melanogaster* (CG). The maximum likelihood tree was calculated on the basis of multiple alignments of the ABC transporter protein sequences. All ABCs were clustered into eight subfamilies (ABCA-H). The green pentagrams represent the genes belongs to the *P. xuthus*, the blue circles indicate the genes among *B. mori*, and the orange boxs show the genes in the genome of *D. melanogaster*. Four Px genes highlighted in grey in ABCG subfamily were selected to investigate their function in the development of *P. xuthus* via CRISPR/Cas9 gene-editing technology

We comprehensively identified copy number of ABC gene family in the genome of the swallowtail butterfly *P. xuthus*. The genome has a total of 56 ABC transporters, which, like those of other insects, is classified into eight subfamilies (A-H) based on the multiple sequence alignment with those of *D. melanogaster* and *B. mori* (Fig. 1; Additional file 1: Table S1 and Table S2). Like that of most other insects, the most expanded subfamily in *P. xuthus* genome is ABCG (30% of total ABC, 17 members), and the next is ABCC (~ 21% of total), while the most expanded subfamily in other arthropods (e.g. Arachnida, Branchiopoda, Copepoda) and even in human is ABCC (Additional file 1: Table S1). These data suggest ABCG may play a more important role in the evolution of diverse insects. All ABC transporter genes of *P. xuthus* vary in length from 1841 bp (*Px_01485_CG10226*) to 33,147 bp (*Px_12497_CG7627*) and each of them possesses at least one nucleotide binding domain (NBD) (Additional file 1**:** Table S2). There are 21 full transporters (each full transporter including two NBDs and two transmembrane domains (TMDs)) in ABCA, ABCB and ABCC subfamilies, 28 half transporters (each half transporter including one NBD and one TMD) in ABCA, ABCB, ABCC, ABCD, ABCG, ABCH subfamilies, and seven atypical transporters (each only including 1 ~ 2 NBD but not TMD). ABCE and ABCF subfamilies contain atypical ABC transporters characterized by a pair of linked NBDs with no TMDs. In addition, three ABC genes (ABCA: *Px_03164_CG32186*; ABCB: *Px_01485_CG10226*; ABCG: *Px_10205_CG11069*) also show ABC domains with only one NBD (Additional file 1: Table S2). Seventeen members of ABCG span in five scaffolds with 2 to 5 genes in each, and 16 of them are typical half transporters, except one with a single NBD (*Px_10205_CG11069*) (Additional file 1: Table S2). Phylogenetic analysis indicates that the four pigmentation related genes (*scarlet*, *white*, *brown* and *ok*), which are all single-copy in *P. xuthus*, form a cluster among three species (*P. xuthus*, *B. mori*, and *D. melanogaster*) (Fig. 1).

**Fig. 2:**
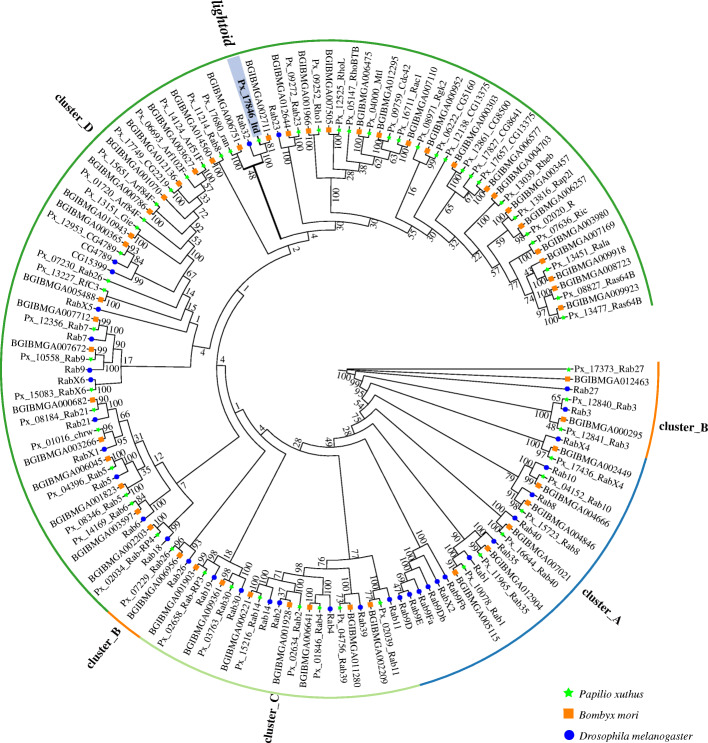
Phylogenetic tree of Rab family of *Papilio xuthus* (Px), *Bombyx mori* (BGIBM) and *Drosophila melanogaster* (CG). The green pentagrams represent the genes belongs to the *P. xuthus*, the blue circles indicate the genes among *B. mori*, and the orange boxs show the gene in the genome of *D. melanogaster*. *Lightoid*, highlighted in red in cluster D, was selected to investigate its function in the development of *P. xuthus* via CRISPR/Cas9 gene-editing technology

We identified 58 and 51 Rab members in the genomes of *P. xuthus* and *B. mori*, respectively (Additional file 1: Table S3), which are nearly twice as much as that in *D. melanogaster* (33) [38] and nematode *Caenorhabditis elegans* (29), but near to that in human (70) [44]. This is the first two cases of genome-wide identification of copy number of Rab gene in lepidopteran insects. Phylogenetic analysis indicates that both genomes showed an expansion of specific-lineage close to clades of Rab32 (*lightoid*) and Rab23 (Fig. 2). Both clades of Rab32 and Rab23 include single-copy orthologs within three investigated species. Among them, *Px_17846_ltd*, together with its ortholog of silkworm (BGIBMGA002711), is single-copy orthologous to *lightoid* of fruit fly (i.e. Rab32), which was found to be essential in eye development, autophagy and lipid storage via vesicle trafficking regulation in *Drosophila* [37, 39] and in silkworm’s response to bacterial challenge [45]. Rab23 is involved in the regulation of the number and planar polarization of the adult cuticular hairs in *Drosophila* [46] and lipid metabolism [39].

### Somatic mutations of four ABCG transporters and one Rab protein in *P. xuthus*

**Fig. 3:**
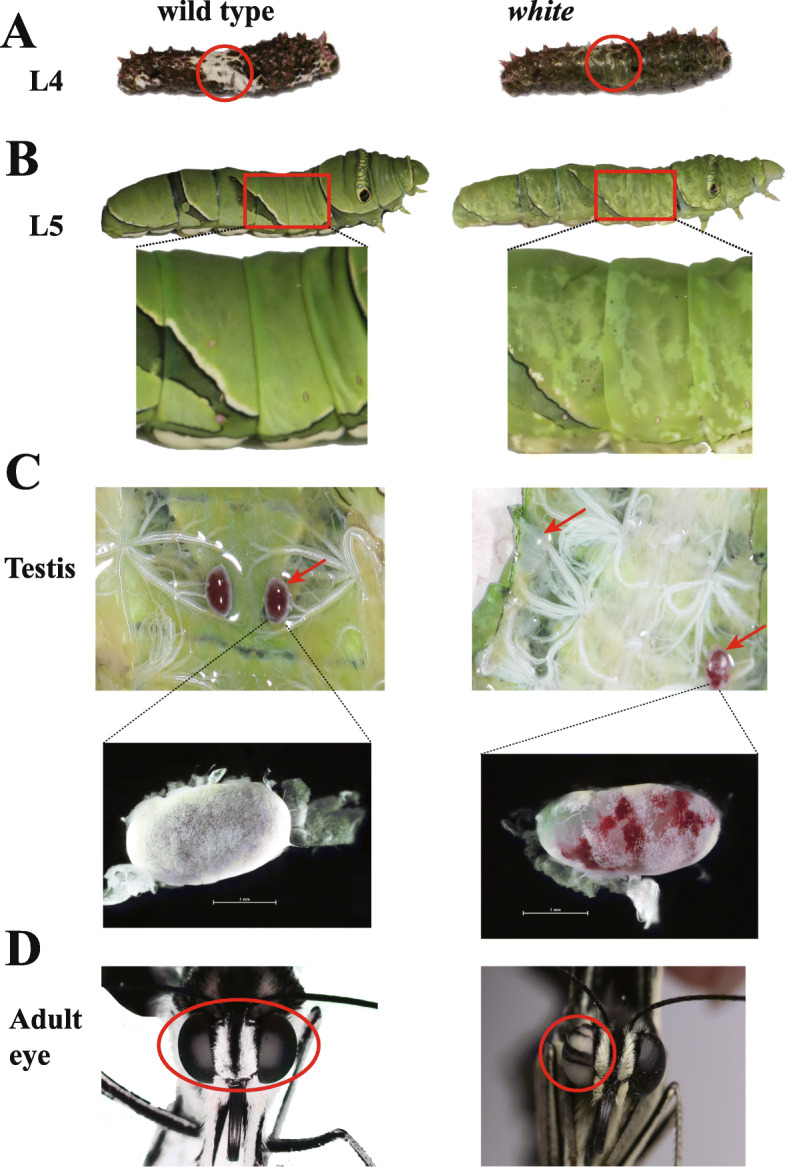
CRISPR/Cas9 disruption of *white* gene resulted in mosaic depigmented phenotypes in larval epidermis, testes and adult eyes of *P. xuthus*. **a** The fourth instar larva (L4). **b** The fifth instar larva (L5). **c** Testes of the fifth instar larva. **d** Adult eyes. Left panel: wild types; right panel: *white* mutants. The area with obviously morphological mutation in mutants and their corresponding part in wild-type were highlighted in red circle in the panels of (**a**) and (**d)** and in red square (**b**). Testes with obviously morphological mutation in mutants and their corresponding part in wild-type were highlighted in red arrow (**c**). Scale bars: 1 mm. The photo credit is provided by Zhiwei Dong

**Fig. 4:**
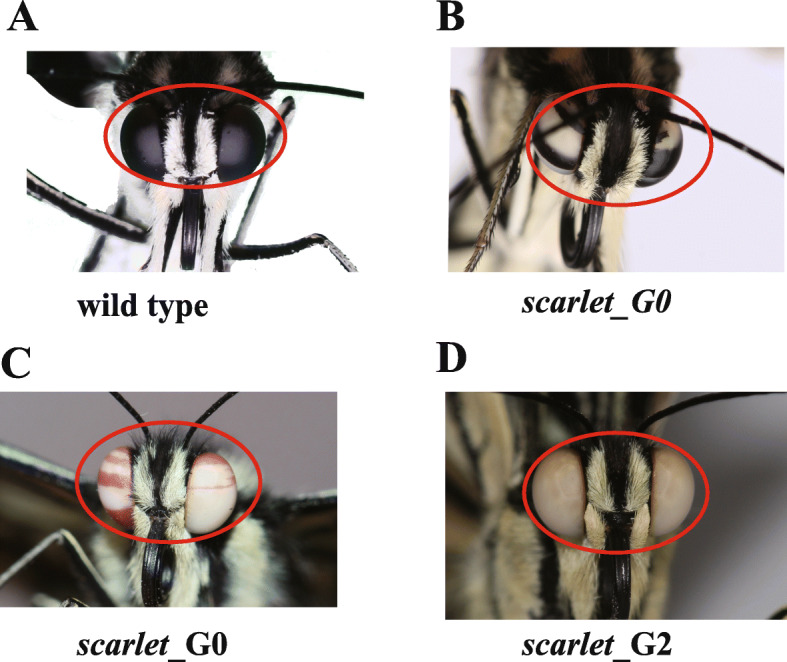
*Scarlet* mutants showed morphological mutation in adult eye color. **a** wild type of adult eyes. **b** G0 (the generation from injected eggs), mutant with white and black mosaic eyes. **c** G0 mutant with red and white mosaic eyes. **d** G2 (the second generation of G0 adults) mutant with white eyes. The photo credit is provided by Zhiwei Dong

**Fig. 5:**
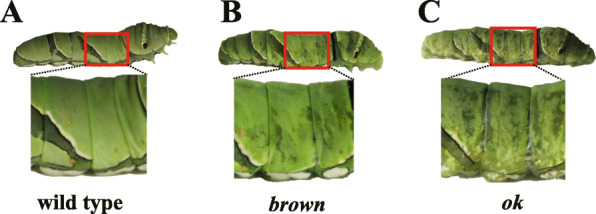
*Brown* and *ok* mutants showed morphological mutation in the fifth instar larva (L5). **a** wild type of L5. **b** *brown* mutant of L5 (**c**) *ok* mutant of L5. The area with obviously morphological mutation in mutants and their corresponding part in wild-type were highlighted in red square. The photo credit is provided by Zhiwei Dong

**Fig. 6:**
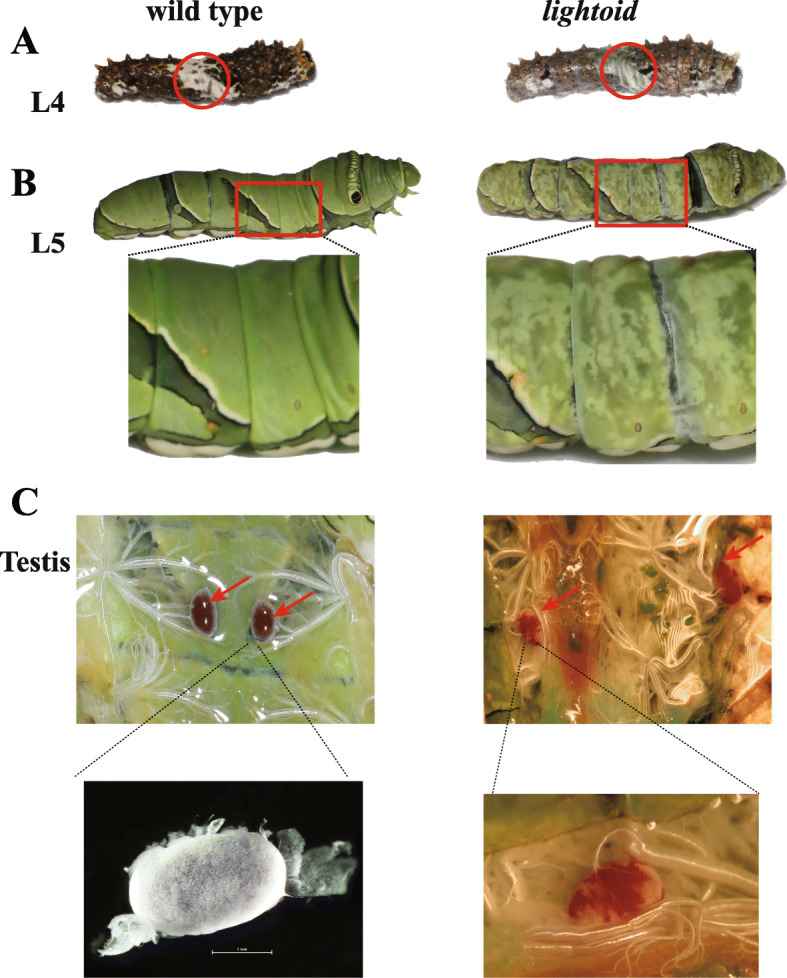
*Lightoid* mutants showed morphological mutations in the fourth instar larvae (L4), the fifth instar larvae (L5) and the testis of L5. **a** L4. **b** L5. **c** Testes of L5. The area with obviously morphological mutation in mutants and their corresponding part in wild-type were highlighted in red circle (**a**) and red square (**b**). Testes with obviously morphological mutation in mutants and their corresponding part in wild-type were highlighted in red arrow (**c)**. Scale bars: 1 mm. The photo credit is provided by Zhiwei Dong

The experiments from *Drosophila* and other insects demonstrate the important roles of five genes (*scarlet*, *white*, *brown*, *ok* and *lightoid*) in pigmentations [12, 18, 19]. However, it is still not sure whether these findings hold for Papilionidae butterflies. To investigate the potential functions of these transporter proteins in swallowtails butterflies, we performed CRISPR/Cas9 gene-editing for these five single-copy genes (*white*, *scarlet*, *brown*, *ok* and *lightoid*) using *P. xuthus* as a model (Tables 1 and 2; Figs. 3, 4, 5, 6; Additional file 1: Tables S4–5; Additional file 2: Fig. S1; Additional file 3: Fig. S2).

**Table 1:** Summary of injected sgRNA and Cas9 mRNA and mutants in CRISPR/Cas9-gene editing experiment. The bracket is the number of larvae and adult which showed phenotypic changes

Gene	Gene ID	Target sites	Final concentration of injected sgRNA (ng/μl)^a^	Injected eggs	Hatching larva (hatching rate)	L5 (Mutants)	Mutation rate in L5 (%)	Pupa	Adult (Mutants)	Mutation rate in adult (%)
*white*	*Px_03417_w*	T_8165, T_8232, T_8700	990	245	76 (31.02%)	69 (21)	30.43	52	39 (5)	12.83
*scarlet*	*Px_03415_st*	T_661, T_684	814	260	48 (18.46%)	28 (0)	0	24	11 (4)	36.36
*brown*	*Px_17845_w*	T_15076, T_16066	925	485	119 (24.54%)	70 (16)	22.86	65	61 (0)	0
*ok*	*Px_17844_st*	T_4354, T_4454	925	250	40 (16%)	12 (3)	25	10	9 (0)	0
*lightoid*	*Px_17846_ltd*	T_2271, T_2307, T_3097, T_3154	800	260	98 (37.69%)	27 (15)	55.55	27	25 (0)	0
Control	NA	NA	NA	31	18 (58.06%)	12	NA	10	10	NA

^a^Cas9 protein concentration is 1000 (ng/μl).

**Table 2:** CRISPR/Cas9 induced phenotype changes of five genes in *Papilio xuthus*

Tissue	Wild-type	***white*** mutant	***scarlet*** mutant	***brown*** mutant	***ok*** mutant	***lightoid*** mutant
The epidermal tissues of the fourth instar larvae (L4)	brownish black integuments with white V-markers	white V-markers change to transparent	NA	NA	NA	white V-markers change to transparent
The epidermal tissues of the fifth instar larvae (L5)	green	transparent mosaic	NA	transparent mosaic	transparent mosaic	transparent mosaic
Testes of L5	red	white, white and red mosaic	NA	NA	NA	white and red mosaic
Eyes of adults	black	white and black mosaic	white and black, pink and white, white	NA	NA	NA
Wings of adults	black and yellow	NA	NA	NA	NA	NA

### Mutations in the *white* gene

We injected the mixed sgRNAs of three target sites (2nd exon: T_8165, T_8232; 3rd exon: T_8700) of *white* gene and Cas9 protein into eggs (Table 1; Additional file 1: Table S4). Compared with wild-types, the edited individuals showed some morphologically changes in both larvae and adults of G0 generation (directly developing from injected eggs) (Fig. 3). In details, the mosaic mutants of the fourth-instar larvae showed a disappearance of V-shaped white markings in their dorsal sides (Fig. 3a), which originally made them mimic to birds dropping to avoid predators. The fifth-instar larvae showed a translucent cuticle instead of green camouflage coloration in wild-types (Fig. 3b). We also observed that the testis of the fifth-instar larval mutants showed part or complete disappearance of white external sheath and red follicular epithelium (Fig. 3c). No changes in shape and color were observed in the pupa and adult wing (Additional file 2: Fig. S1B). Some of adults developed from larval mutants showed abnormal eyes with white and black mosaic color stripes instead of black eyes in wild-types (Fig. 3d).

### Mutations in the *scarlet* gene

We injected the mixed sgRNA of two target sites (2nd exon: T_661, T_684) of *scarlet* gene and Cas9 protein into eggs (Table 1; Additional file 1: Table S4). No morphological changes were observed in the injected G0 larvae, but 36.36% (four individuals: three females and one male) emerged adults of G0 showed abnormal eyes with mosaic stripes of white and black/red-brown (Table 1 and Fig. 4b, c), but their wing pattern show no changes (Additional file 2: Fig. S1C). Because of the discordance of emergence time for male and female mutants, we made a cross of a wild-type female (F_wt_) adult with G0 male adult mutant (M_G0_) of mosaic white and red-brown eye color and get six G1 adults, for which no morphological change was observed. We further made a cross between G1 female and male adults (F_G1_, M_G1_) to obtain four G2 adults (one female and three males), all of which showed the complete white eyes (Fig. 4d).

### Mutations in the *brown* and *ok* genes

We injected the mixed sgRNA of two target sites (3rd exon: T_16066; 5th exon: T_15076) of *brown* gene and Cas9 protein into eggs (Table 1; Additional file 1: Table S4). We observed that 22.86% of the fifth-instar larvae of G0 showed a translucent cuticle (Table 1 and Fig. 5b), similar to that of *white* mutants. However, unlike those of *white* mutants, mutated fifth-instar larvae of *brown* have normal testis, and all mosaic G0 adults have normal black eyes and wing (Additional file 2: Fig. S1D). We also injected the mix sgRNA of two target sites (3rd exon: T_4354, T4454) of *ok* gene and Cas9 protein into eggs (Table 1; Additional file 1: Table S4). Similar to that of its close paralog *brown* mutants, G0 fifth-instar larvae of *ok* also showed a translucent cuticle (Fig. 5c), but normal testis and normal wing pattern (Additional file 2: Fig. S1E), and the mosaic G0 adults also have normal black eyes.

### Mutations in the *lightoid* gene

We injected the mixed sgRNAs of four target sites (2nd exon: T_2307, T_2271; 3rd exon: T_3154, T3097) and Cas9 protein into eggs (Table 1**;** Additional file 1: Table S4). Like that of *white* disruption, we observed the disappearance of V-shape white markings in the fourth-instar larvae of G0 (Fig. 6a) and a translucent cuticle in their fifth-instar larvae (Table 1, Fig. 6b), but the adult wing pattern is unaffected (Additional file 2: Fig. S1F). Anatomy of these mutated fifth-instar larval testis also showed partially disappearance of white external sheath and red follicular epithelium (Table 1, Fig. 6c), just like that of *white* mutants. But unlike *white* mutants, no morphological changes were observed in G0 adults of *lightoid* developed from the fifth-instar larval mutants.

### Genotyping of mutants

Genomic DNA was isolated from mutant adults/larvae, and PCR amplicons including the region of target sites were cloned and sequenced. The sequenced data validated that these five genes were disrupted in their corresponding mutants (Additional file 1: Table S5; Additional file 3: Fig. S2). All six G0 mutants of *white* (three 5th-instar larvae and three adults) showed the disruption (10–100% mutated rate) in all or part of target sites with numerous deletions (1–84 bp), inserts (1–30 bp) or substitutions in the targeted regions (Additional file 3: Fig. S2A). Four G2 adult mutants of *scarlet* showed a deletion of 8–11 bp in the target site T_684 in all clones (Additional file 3: Fig. S2B), suggesting that these G2 adults may be homozygous mutants of *scarlet* locus. All G0 larval mutants of *brown* were disrupted (mutated rate: 80–100%) in two target sites (T_15076 and T_16066) with numerous deletions (1–52 bp), inserts (2–21 bp) or substitutions (Additional file 3: Fig. S2C). All three larval mutants of *ok* were disrupted in target sites T_4354 and T_4454 with numerous deletions (2–25 bp), inserts (3–8 bp) or substitutions (Additional file 3: Fig. S2D). All three larval mutants of *lightoid* gene showed numerous deletions (1–24 bp), inserts (3–25 bp) or substitutions in all or part of target sites (T_3154, T_3097, T2307 and T_2271) (Additional file 3: Fig. S2E).

### Transcriptome profiling of the mutants

To further investigate transcriptomic profiles involved with these pigment-related transporters, we dissected the epidermal tissues of the fifth-instar larval mutants induced by the disruption of *white*, *brown*, *ok* and *lightoid* genes and head tissues of adult mutants induced by the disruption of *scarlet* gene for transcriptomic sequencing. In total, 172 Gbp transcriptomic data and average 51 M reads per library were generated for 22 individuals (Additional file 1: Table S6), which are verified to be mutated at genomic DNA level. The average mapping depth of RNA reads in exon regions varied from 125× to 204× with the reads alignment ratio varying at 83.56–90.80% for both mutants and wild-types (Additional file 1: Table S7), suggesting that the transcriptomic data is adequate for transcriptomic analysis and identification of differentially expressed genes (DEGs) between mutants and wild-types.

### Variations in transcripts in mutants of five disrupted pigment transporting genes

**Fig. 7:**
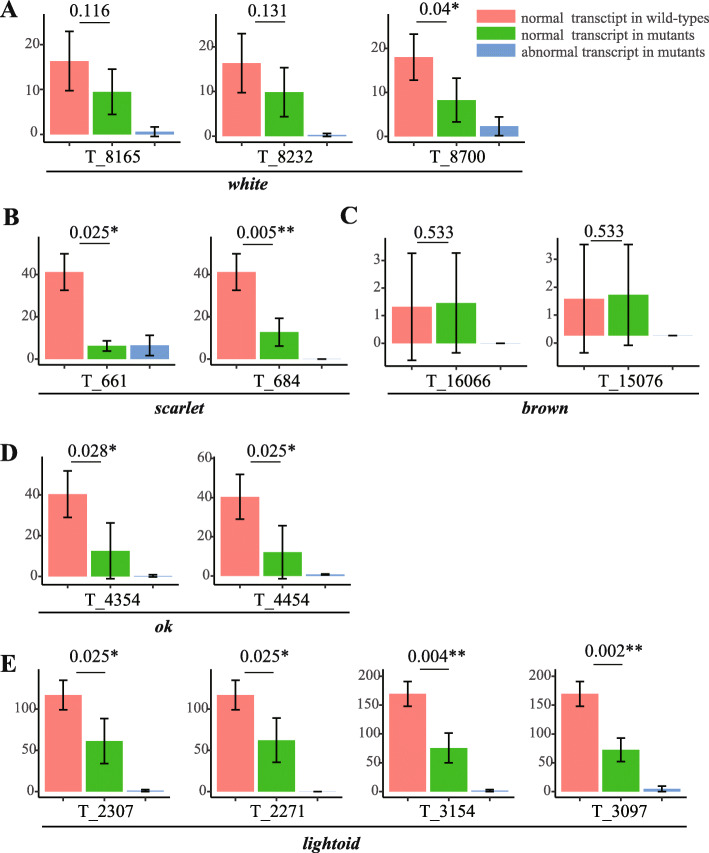
Expression level of the exons in which the target sites are located. **a** *Px_03417_w* (*white*). **b** *Px_03415_st* (*scarlet*). **c** *Px_17845_w* (*brown*). **d** *Px_17844_st* (*ok*), and (E) *Px_17846_ltd* (*lightoid*). Red, green and blue color indicates the expression level of normal transcripts in wild-types, normal transcripts in mutants and abnormal transcripts in mutants, respectively. The expression level is evaluated by FPKM (Fragments Per Kilobase of exon model per Million mapped reads). The number and marker above the line is the *P*-value, which is performed with *t*-test and are marked with * (less than 0.05) and ** (less than 0.01), respectively

The analysis of the transcriptomic sequencing depth indicate that most mutated individuals showed a deletion of several bases or reduced mapping depths in target regions than those of wild-types (Additional file 4: Fig. S3). Further analysis of nuclear variant calling (including SNPs and INDELs) for all the samples confirmed INDELs in the transcripts of most target regions, and also identified some SNP mutation in the regions of some targets (Additional file 1: Table S8). Specifically, a homozygous 8-bp deletion was identified at the region of target site T_684 in the transcripts of four investigated *scarlet* mutants of G2 (Additional file 1: Table S8; Additional file 4: Fig. S3B), as shown in PCR genotyping (Additional file 3: Fig. S2B). For G0 mutants of other four genes (*white*, *brown*, *ok*, *and lightoid*), a deletion of several bases or reduced mapping depths in target regions can be detected (Additional file 4: Fig. S3A, C, D, E). To further explore how the mutations introduced by CRISPR/Cas9 gene-editing affect the expression of the genes, the expression level (Fragments per Kilobase Million, FPKM) of the exon involved with target sites were acquired by manually distinguishing the mutated reads and normal reads in the mutant samples (Fig. 7). Our data indicated that except T_16066 and T_15076 of *brown*, the exons of all other target sites showed a lower expression in mutated individuals than in wild-type individuals. Among them, the exons of most target sites (excl. T_8165 and T_8232 of *white*) showed a significantly (*t*-test, *P*-value < 0.05) decreased expression of normal transcript in mutated individuals than in wild-type individuals (Fig. 7a, b, d, e), suggesting that the normal transcripts were less transcribed after CRISPR/Cas9-induced mutations, thus leading to the down-expression of the five genes. For T_16066 and T_15076 of *brown*, they showed a slightly higher expression of normal transcripts in mutant samples than in wild-type samples (Fig. 7c), which may be caused by the dosage compensation [47]. In summary, these transcriptomic data demonstrated that mutations induced by CRISPR/Cas9 at genomic level can produce abnormal expression with accumulation of abnormal transcripts and decrease or dosage compensation of normal transcripts at transcriptomic level.

### Differentially expressed genes (DEGs) and their functions analysis

**Fig. 8:**
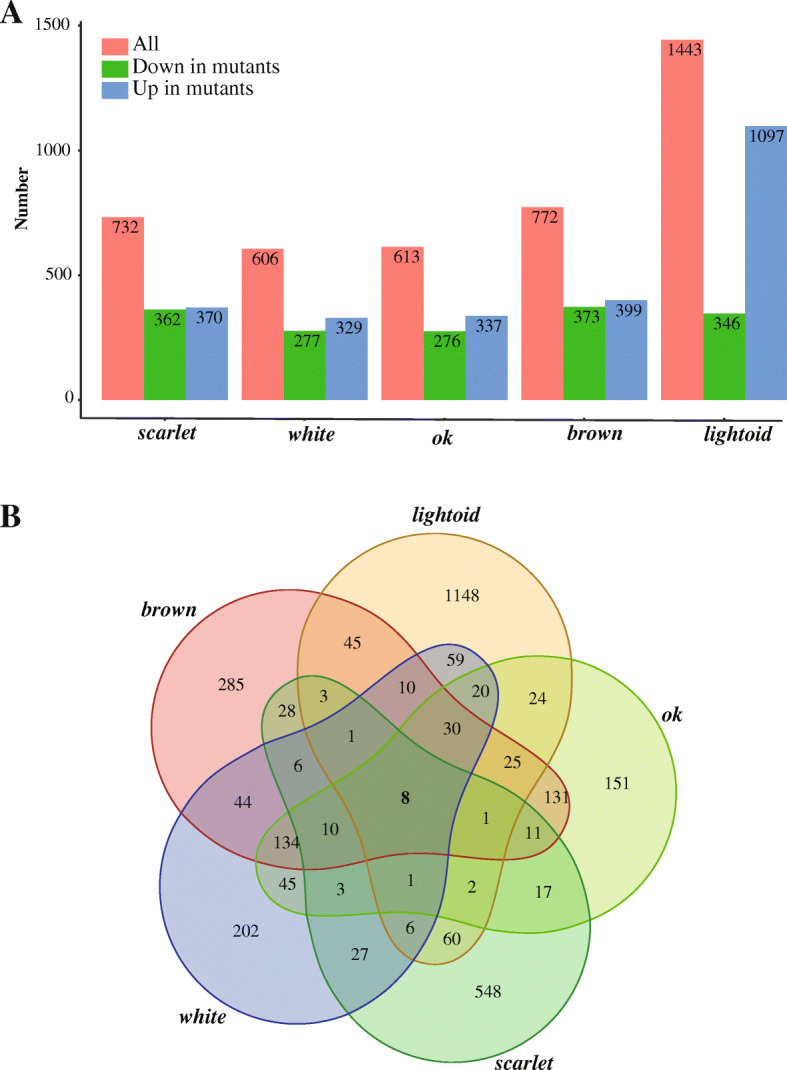
The number of differentially expressed genes (DEGs) among mutants of the five genes and their Venn diagram. **a** The number of DEGs between mutants of the five edited genes and their wild-types. **b** Venn diagram of DEGs of mutants of the five edited genes and their wild-types

Both the correlation analysis (Additional file 5: Fig. S4A) and the principle component analysis (PCA) (Additional file 5: Fig. S4B) based on transcriptomic data among individuals of mutants and wild-types showed that mutants and wild-types form two separate clusters, suggesting a high correlation of expression among mutants or wild-types. The number of DEGs among heads of G2 *scarlet* mutants and wild-types is 732 with half down-expressed (362) and another half up-expressed (370) (Fig. 8a). The epidermis of the fifth-instar larvae among mutants of *white*, *brown*, *ok*, and *lightoid* and their wild-types have 606, 772, 613 and 1443 DEGs, respectively (Fig. 8a). Among them, up-expressed DEGs of *white* (329), *brown* (399) and *ok* (337) mutants are a little more than their down-expressed DEGs, while *lightoid* mutants has about a three folds up-expressed number of DEGs (1097) than down-expressed (346). We found that mutants of *scarlet*, *white*, *brown*, *ok* and *lightoid* shared eight DEGs (three genes down-expressed in all mutants: *Px_02773_Cyp6d4*, *Px_13524_CG10175*, *Px_15008_CG9701*; four up-expressed in all mutants: *Px_00724_unknow*, *Px_00828_amx*, *Px_02067_unknow*, *Px_03043_unknow*; one up-expressed in *scarlet* mutants but down-expressed in other mutants: *Px_03657_ImpL2*) (Fig. 8b and Additional file 1: Table S9), suggesting some intersections in the expression profile of these transported-related genes. We found 10 DEGs shared in the mutants of all four ABC transporter and another 30 DEGs shared among the fifth-instar larval mutants of four gene *white*, *brown*, *ok* and *lightoid* (Additional file 1: Table S9).

**Fig. 9:**
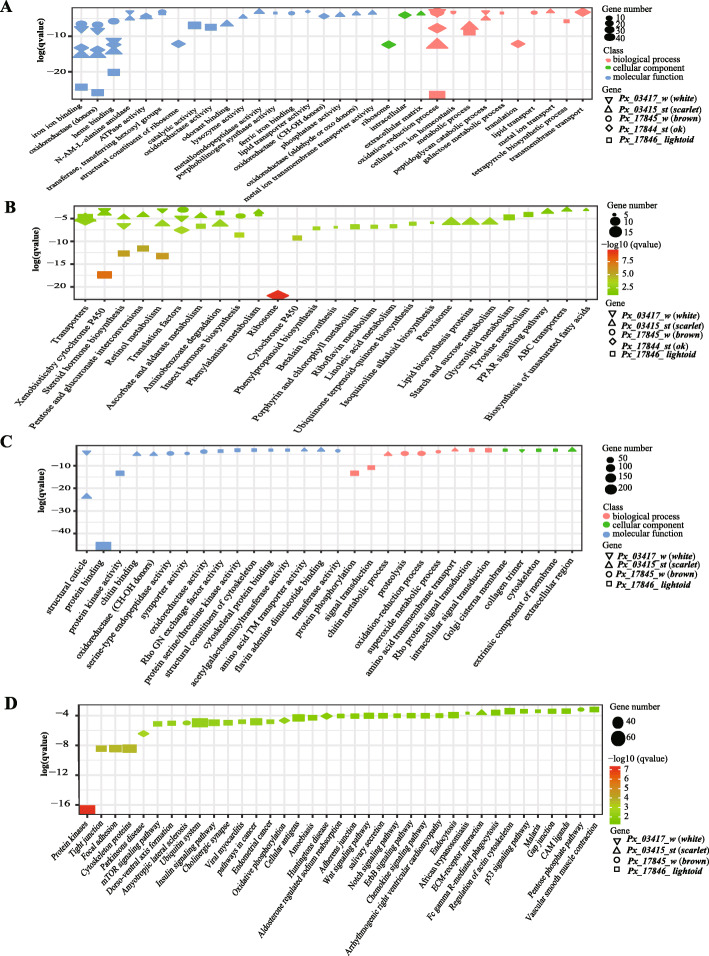
The functional enrichment of Gene Ontology (GO) term and KEGG pathway for the differentially expressed genes (DEGs) which were down and up expressed in the mutated groups. **a** and **b** represent the gene enrichment of GO term and KEGG pathway of down-regulated DEGs, separately. **c** and **d** represent the gene enrichment of GO term and KEGG pathway of up-regulated DEGs, separately

Against annotated genes of *P. xuthus* genome with GO and KEGG annotation, we performed enrichment analysis on all DEGs of mutants. GO enrichment analysis show most DEGs in mutants of all five genes enriched in the molecular function categories and biological process (Fig. 9a, c and Additional file 6: Fig. S5A), though no shared patterns among them were identified in KEGG enrichment (Fig. 9b, d and Additional file 6: Fig. S5B). DEGs of four ABCG transporter (*brown*, *ok*, and *white*, *scarlet*) mutants shared four GO terms, including iron ion binding (GO: 0005506) and heme binding (GO: 0020037) and oxidoreductase activity (GO: 0016705) in molecular functions, and oxidation-reduction process (GO: 0055114) in biological process. DEGs of *brown*, *ok*, and *white* fifth-instar larval mutants enriched in the KEGG pathway of transporters. Most DEGs of *lightoid* were enriched in glycoprotein binding (GO: 0005515), and others enriched in protein kinase activity (GO:0004672), protein phosphorylation (GO:0006468) and signal transduction (GO:0007165); and the most conspicuous enriched KEGG pathways of *lightoid* are protein kinases, tight junction, focal adhesion, cytoskeleton proteins and amoebiasis. We also found some DEGs of *scarlet* mutants are specifically enriched in structural constituent of cuticle (GO:0042302), chitin binding (GO:0008061), chitin metabolic process (GO:0006030), lysozyme activity (GO:0003796), phosphatase activity (GO:0016791) in GO analysis, and in many KEGG pathways such as lipid biosynthesis, phenylalanine metabolism, tryptophan metabolism and amino acid related enzymes etc.

## Discussion

We used CRISPR/Cas9-based mutagenesis to uncover the roles of four ABCGs (*white*, *scarlet*, *ok*, and *brown*) and one Rab member (*lightoid*) in the morphological development of swallowtails butterfly for the first time. Our experimental data demonstrated that all these genes contributed to morphological development of larvae (cuticle, testis) and/or adult eyes in swallowtail butterfly. *White* play a key role in the morphological development of both larvae (cuticle, testis) and adults (eye color), while other four transporters play an important role in larvae (cuticle: *brown*, *ok*, *lightoid*; testis: *lightoid*) or adult eye color (*scarlet*). Especially two genes (*white*, *lightoid*) were for the first time discovered to contribute larval testis. Combining the results from swallowtail butterfly and other insects, we found that all these transporters, though as orthologs or paralogs, show some lineage-specific phenotypes (Additional file 1: Table S10). Like in *P. xuthus*, *white* was found to contribute both adult eye color and larval cuticle by affecting tryptophan, guanine and uric acid transport in other lepidopteran insects including Satyrinae butterfly *B. anynana* [35], silkworm *B. mori* [9], and cotton bollworm *H. armigera* [10]. Nevertheless, it was reported to contribute only adult eye colors in other non-lepidopteran insects such as Diptera (*Anopheles gambiae*, *A. albimanus*, *Aedes aegypti*) [5, 48], Coleoptera (*Harmonia axyridis*, *T. castaneum*) [8, 49], Hemiptera (*Nilaparvata lugens*, *Lygus hesperus*, *Limnogonus franciscanus*) [32, 33], Orthoptera (*Acheta domesticus*), and in Crustacea *Daphnia magna* [31] (Additional file 1: Table S10). Lack of *white* protein was recessive embryonic lethal in *H. armigera* [10]. *White* and *scarlet* were verified to be heterodimer and function as a full transporter to transport tryptophan metabolites in ommochromes pathway in fruit fly [11, 26]. Thus, the mutant of both *white* and *scarlet* showed white eyes in *D. melanogaster* because of failure to transport either guanine or tryptophan into pigment cells [11, 28]. Especially, homologous disruption of *scarle*t in G2 adult result in white eyes, suggesting only ommochromes contributing to *P. xuthus* eye color, which is consistent with its function in silkworm [30]. The disruption of *scarlet* mainly displayed the mutated phenotype of adults’ eyes in insects including Lepidoptera (butterflies: *P. xuthus* (this study), *B. anynana*; moths: *B. mori*, *H. armigera*) and non-lepdopteras (Diptera: *D. melanogaster*; Coleoptera: *T. castaneum*, *H. axyridis*; Hemiptera: *N. lugens*, *L. hesperus*) [7, 9, 10, 28, 32–35, 49], and in crustacean (*D. Magana*) [31], but it also affect larval cuticle in one moth (*H. armigera*) [10]. The *brown* and *white* form heterodimer and transport the pteridines precursor into the pigment granules in the eye development of *D. melanogaster* [50]. It was reported that disruption of *brown* and *white* would affect the eye development of other two hemipteran insects (*Lygus hesperus*, *Nilaparvata lugens*) [32, 33]. However, it only affects larval development in butterfly (*P. xuthus*: larval cuticle) (this study) and in silkworm (acting as a riboflavin transporter in Malpighian tubule) [51]. *Ok*, a paralog of *brown* and identified only lepidopteran, can incorporate uric acid into the epidermis by forming heterodimers with white protein, and thus its mutants in moths show a translucent, oily-appearing epidermis in larval skin [3]. It showed effects on only larval cuticle of butterfly *P. xuthus*, but on both larval cuticle and adult eyes of another moth *H. armigera* [10]. Unlike in *D. melanogaster,* Rab-RP1 (*lightoid*) didn’t contribute to eye color of adult but only larval cuticle and testis in butterfly development. Rab-RP1 (*lightoid*) affected adult eye color by participating in biogenesis or degradation of pigment granules in *Drosophila* [36, 37]. In silkworm, BmRABRP (*lightoid*) mRNA and protein were found to be high expressed in the Malpighian tubule and fat body, respectively, and played an important role in the response to bacterial challenge [45]. Rab proteins are the largest branch of the Ras-like small GTPase superfamily. They vary between GTP- and GDP-bound states, which are facilitated by guanine nucleotide exchange factors (GEFs) and GTPase-activating proteins (GAPs), and function as molecular switches in transporting regulation of intracellular membrane trafficking in all eukaryotic cells [52]. Combining with our finding that Lepidopteran insects have an expansion of specific-lineage close to clades of Rab32 (*lightoid*) and Rab23 (Fig. 2), we speculate that Rab32 (*lightoid*) have gained new function, while other Rab copies may perform the transportation of pigment-related granules.

Unexpectedly, our experimental data demonstrated that all these genes didn’t contribute to wing color of swallowtail butterfly. These findings suggest that these investigated transporters, contrary to the postulated in previous works [2, 53], do not take part in transportation of the tryptophan-derived metabolite kynurenine, which is one of precursors in biosynthesis of papiliochrome [17]. Another possibility is that the five edited genes do have result in the reduced transportation of kynurenine but other genes may also transport kynurenine into wings and rescue the low concentration of kynurenine, finally leading to unchanged color in their wings. The current study investigated only some gene copies of pigment-related G subfamily of ABC transporters and the cluter_D group of Rab family (Figs. 1 and 2). Further experiments should be carried out to investigate the function of other transporters G subfamily of ABC transporters and the cluter_D group of Rab family, which may include the candidates to play a role in biosynthesis of papiliochrome.

Characterization of transcriptome can help explain the functional complexity of these genes. We noticed the mutants of all five genes have eight shared DEGs, suggesting some intersections in the expression profiles of these transported-related genes (Additional file 1**:** Table S9). Three down-expressed DGEs (*Px_02773_Cyp6d4*, *Px_13524_CG10175*, *Px_15008_CG9701*) in all five mutants are involved with detoxification. *Px_02773_Cyp6d4* belongs to cytochrome P450 family, which is related to detoxification of such chemicals as pyrethroids but is not critical for the metabolism of vital endogenous substrates in fruit fly [54, 55]. However, our result suggests that the *cyp6d4* gene is possibly involved in oxidation-reduction reaction of substances related to pigment metabolism and synthesis in butterfly *P. xuthus*. *Px_13524_CG10175* is annotated as carboxylesterase, which is one of important detoxification systems [56–59]. *Px_15008_CG9701* is annotated as lactase-phlorizin hydrolase, which plays important roles in locust detoxification [60, 61]. In *Drosophila*, kynurenine pathway is centrally related to toxicity because its intermediate 3-hydroxykynurenine (3-HK) can generate free radicals by auto-oxidation [62]. In insects, the ommochromes pathway is also the most important route for elimination of tryptophan metabolites, which are toxic in the presence of excessive quantities [63]. On the other hand, the 3-HK and kynurenine are also precursor of ommochromes pathway [64]. Thus, the pigmentation process may be one form of detoxification process. Among four up-expressed genes of DEGs in all mutants, three (*Px_00724_unknow*, *Px_02067_unknow*, *Px_03043_unknow*) show unknow function against fly *Drosophila melanogaster* annotation result; another one (*Px_00828_amx*) is annotated as TM2 domain-containing protein *almondex*, which is involved in several processes such as ectodermal cell fate determination, lateral inhibition, and positive regulation of notch signaling pathway [65]. In *D. melanogaster*, the *almondex* (*amx*) plays roles not only in the embryo but also in imaginal specification of the eyes [66]. *Px_03657_ImpL2*, the gene up-expressed in *scarlet* mutants but down-regulated in other mutants, is annotated as Neural/ectodermal development factor ecdysone-inducible gene L2 (*ImpL2*). In fruit fly, *ImpL2* can reduce systemic insulin/IGF signaling and causes systemic organ wasting [67], extend the lifespan [68], and affected eye development by regulating one jak and one stat (stat92E) [69]. Our result confirm that *ImpL2* are also related to eye development in butterfly. Ten DEGs and some enriched GO items were shared in the mutants of four ABCG members (*white*, *scarlet*, *brown* and *ok*) suggesting these homologs shared some common molecular basis in different developmental stages of different mutants (Additional file 1**:** Table S9). Among them, iron ion binding (GO: 0005506) and heme binding (GO: 0020037) may play an important role in color display [70, 71], while oxidoreductase activity (GO: 0016705) and oxidation-reduction process (GO: 0055114) may be related to the synthesis of related compounds [72–74]. Among shared DEGs, one gene (*Px_10696_CG1640*) was down-expressed in all four ABC mutants and annotated as amino transferases, which play important role on amino acid metabolism. One up-expressed DEG (*Px_08852_Cpr56F*) is annotated insect cuticle protein, which is important constituent of insect cuticle. Among four ABC mutants, *scarlet* is eye mutants of G2 adult and other three (*white*, *brown*, *ok*) are cuticle mutants of the fifth-instar larvae. We notice some differences of DEGs between eye mutants and larva mutants. One DEG (*Px_03657_ImpL2*, annotated as Neural/ectodermal development factor) shared in all five mutants and one DEG (*Px_05180_Cyp9f2*, annotated as Cytochrome P450) shared in four ABC transporter, showed up-expression only in *scarlet* mutants; on the other hand, similar genes were enriched in the pathway of transporters in the fifth-instar larval mutants of *brown*, *ok*, and *white*. *Lightoid* had similar larval phenotypes to those of *white*, *brown* and *ok*, but no shared GO and KEGG enrichment were identified among DEGs of *lightoid* and any other ABC transporters. In contrast, DEGs of *lightoid* mutant was related to glycoprotein binding, protein kinase activity and protein phosphorylation, signal transduction, tight junction, focal adhesion, cytoskeleton proteins, amoebiasis. Regardless of no shared function enrichment of DEGs among mutants of *lightoid* and other ABC transporter, 30 DEGs were identified to be shared among the fifth-instar larval mutants of four genes (*lightoid*, *white*, *brown*, and *ok*) (Additional file 1**:** Table S9). Among them, five P450 genes, one member of major facilitator superfamily is included among 17 down-expressed DEGs, while G-protein, laccase, ABCC and apterous are among 13 up-expressed DEGs. These results suggest that *lightoid* plays a role in signal transmission mainly through the phosphorylation cascade in the process of pigment transportation. Besides, our findings also provide some insights into genotyping-phenotyping sequencing profile in gene-editing study of especially non-model animals. In most cases, mosaic mutants may be used to check the function of genes in non-model animals because it is hard to get homozygous offspring of mutants. In such cases, we should remind that although the gene could be disrupted by high mutated rate at DNA level, different expression profiles of target gene could be observed. It is thought that after disrupted, genes will show decreased expression [2, 75–77]. Our results show that is not always the truth for mosaic mutants which include both disrupted and normal tissue. In this study, most mutants show a lower expression; however, although high mutated rates were observed for *brown* gene at DNA level (80–100%) (Additional file 1: Table S5), its mutants show even a little higher transcriptomic expression than wild-type (Fig. 7c), which is likely to be affected by the dosage compensation effect of gene. The similar phenomenon was also reported in mosaic mutants of homeobox gene *abdominal B* (*Abd-B*) disruption of one firefly [78]. These data provide evidence that a mosaic of phenotype change in mosaic mutants result from the fraction of abnormal transcript in altered tissues and normal transcripts in unaltered tissues.

## Conclusions

For the first time, we comprehensively identified copy number of ABC family (56) and Rab family (58) in the genome of the swallowtail butterfly *P. xuthus*. We investigated the roles of four ABCGs (*white*, *scarlet*, *brown*, and *ok*) and one Rab gene (*lightoid*) in *P. xuthus* development using CRISPR/Cas9 gene-editing technology. The results indicated that all these five genes play an important role in the morphological development of larvae (cuticle and/or testis) and/or adults’ eye color, but have no effect on wing color. Comparative transcriptomes of mutants and wild-types revealed some molecular mechanisms of these genes commonly or specifically underlying their phenotypic traits. Further functional verification on paralogs of ABCG and Rab, especially those members phylogenetic close to those here investigated functionally, may provide more evidence of body color in butterflies.

## Methods

### Gene identification, sequence alignment and phylogenetic analysis

To identify genes encoding ABC transporters, BLASTP searches (E-value < 10^− 5^) were performed against *P. xuthus* genome [2] using the reported ABC protein sequences of *D. melanogaster* and *B. mori* [22, 79] as queries. The conserved nucleotide binding domain (NBD, PF00005.24) and transmembrane domain (TMD, PF00664.20) were scanned for putative ABC transporter genes using the Hidden Markov Model (HMM) HMMER v3.2.1 [80]. After removing redundancy, putative transporter genes with the best hit score were retained as candidate ABC genes. To assign the candidate ABC genes into different subfamilies, multiple alignments of the ABC transporter protein sequences were performed using MAFFT [81], and the poor aligned regions and partial gaps were removed with trim-AI (gt =0.5) [82]. Then, the alignments were subjected to a phylogenetic analysis using RAxML [83] based on the Maximum Likelihood (ML) method with settings “-f a -x 12345 -N 100 -p 12345 -m PROTGAMMAWAG”. The resulting trees were displayed and edited using interactive tree of life (iTOL) v3 [84]. The subfamily assignment of ABC proteins in each species was further confirmed using BLASTP analyses at the NCBI webserver (www.ncbi.nlm.nih.gov/blast).

To identify Rab family in *P. xuthus* and *B. mori* genomes, respectively, BLASTP searches (E-value < 10^− 5^) were performed against the *P. xuthus* and *B. mori* genomes using the Rab protein sequences of *D. melanogaster* (http://flybase.org/). We filtered out the genes with identities lower than 30%. Two conserved domains (Ras: PF00071.19 and Roc: PF08477.10) were scanned by the Hidden Markov Model (HMM) HMMER v3.2.1 [85]. After removing redundancies, top hits for putative genes were retained. As the methods used in ABC transporter classification, we also performed sequence alignments and phylogenetic analysis with the same software and parameters, except for removing gaps (gt = 0.2).

### CRISPR/Cas9 induced mutation

#### SgRNA design and synthesis

The sgRNA target sites were designed based on principle 5′-**N**_**20**_NGG-3′ (Additional file 1: Table S4) [86]. The dsDNA template for sgRNA production was generated according to our previously protocol [2, 75]. Recombinant Cas9 protein (PNA Bio Inc., CA, USA) were purchased directly.

#### Egg collection, microinjection, breeding and phenotyping

Eggs were collected within 30 min after lay and placed on a microscope slide and fixed by glue. Microinjection were performed with final concentrations of 1000 ng/μl Cas9 protein and 800–990 ng/μl sgRNA (Table 1) using a TransferManNK2 and FemtoJet microinjection system (Eppendorf, Hamburg, Germany). All operations were finished within 2 h after lay. Approximately 245–485 eggs (Table 1) were injected for each gene and incubated at the condition with 25 °C, 12 h light/12 h darkness, 80% relative humidity and darkness for 4–6 days until hatching. Hatched larvae were transferred to host plant leaves (*Zanthoxylum piperitum*) for breeding and reared at 27 °C, 16 h light/25 °C, 8 h darkness and keep 80% relative humidity. The morphological changes were observed mainly from the fourth-instar larvae in order to avoiding the disturbance on early young larvae (first to third instar) which have similar morphology to the fourth-instar larvae. Pupae were transferred into plastic baskets before eclosion. Emerged adults were crossed via hand pairing, and then mated females were placed in net rooms with host plants for oviposition.

#### Genomic DNA extraction and mutagenesis detection

Part epidemic tissues of the fifth-instar larval mutants of four genes (*white*, *brown*, *ok*, *lightoid*) or the thorax and abdomen of adults (*white*, *scarlet*) and their corresponding wild types were dissected in phosphate buffer saline and then used to extract genomic DNA using TreliefTMAnimal Genomic DNA Kit (TsingKe, China) following the manufacturer’s protocols. The tissues of each individual were as a biological sample. At least three replicates were carried out for mutants of each gene. Except some adult mutants of *white* gene, part tissues of the same individual for the mutants and wild-types are also used for RNA extraction as described in the following part. Subsequently, primers flanking the target sites for each gene (Additional file 1: Table S4) were designed, and the PCR reaction were carried out using the 20 μl volumes, according to TransDirect PCR SuperMix (Trans, China). PCR products were TA-cloned into PMD19 vectors (Takara, Japan) and 10 clones were randomly picked up and sequenced for each individual. Sequence data were analyzed using SeqMan software (DNASTAR7.0) to determine the exact mutation type.

#### Transcriptome sequencing and data analysis

Part epidemic tissues of the fifth-instar larvae for the mutants of four genes (*white*, *brown*, *ok*, *lightoid*) and wild types or the head tissues of adult for mutant of *scarlet* gene and wild types were dissected for RNA extraction and sequencing. These individuals are the same as those used in genotyping above mentioned. The tissues of each individual were as a biological sample. At least three replicates were carried out for mutants of each gene and their wild-types, and total 22 samples from 22 individuals were included (Additional file 1: Table S6).

Total RNA was isolated using TRIzol reagent (Invitrogen, USA) according to the manufacturer’s instructions. The 350 bp insert size paired-end libraries were generated using Illumina mRNA-Seq Prep Kit and were sequenced using Illumina HiSeq4000 sequencers with read length of PE150 at Novogene (Tianjin, China). After removing adapter sequences, about 6 Gbp raw reads were generated for each sample. The quality of the reads was evaluated using FastQC (https://www.bioinformatics.babraham.ac.uk/projects/fastqc/). We also filtered out those reads with more than 10% Ns or more than 30% low-quality bases (base quality < 20) using a custom Perl script. All cleaned reads were mapped back to the assembled genome of *P. xuthus* [2] using hisat2 [87] with the default parameters. After the reads were mapped, SortSam program of software Picard v2.18.9 (https://broadinstitute.github.io/picard/) was used to convert the sam file into bam file and sort according to the coordinate. HTSeq v0.11.2 [88], a python package used to analyze high-throughput sequencing data, were used to count the number of reads mapped on each gene. About the average depth of exons, we employed Samtools v1.3.1 [89, 90] to calculate the mapping depths of exonic regions and the average depth of the bases located in those exons was treated as the average mapping depth for exons. Then we manually count the number of the mutated reads which cover in the target sites for each sample, calculate the expression level (FPKM) of mutated transcripts and normal transcript in CRISPR-indued mutated individuals for exons with target sites. Further, *t*-test is used to test whether the expression level of normal transcripts is significantly decreased in CRISPR-induced mutant samples.

We also used a R package named DESeq2 [91] with the default pipeline to normalize the expression level to reduce the bias due to different amplification during PCR and calculate the expression level. Then clustering and principal component analysis (PCA) were done based on data that has suffered variance stabilizing transformation with *vst* function in DESeq2 [91]. Both the clustering and PCA showed clear separation between the unedited and edited individuals. Therefore, we used *results* function to identify the differentially expressed genes (DEGs) (|log_2_FC| ≥ 1 && FDR < 0.05). The GO and KEGG enrichment analysis of DEGs were then performed via InterProScan 5 [92] and BLASTP v2.4.0 [93] based on a custom R script with hypergeometric test, respectively. The *P*-values were corrected with Benjamini-Hochberg FDR. SNP calling analysis followed the process of hisat2/SortSam/MarkDuplicates (Picard)/Samtools/Bcftools v 1.3.1 [89, 90] . The genotypes with allele depth less than 5 (AD < 5) and genotype quality less than 20 (GQ < 20) were treated as missing genotypes. The mutations with missing genotypes in all mutant samples were removed. In addition, the mutations only identified in wild-type individuals also were removed because these mutations were not caused by CRISPR gene-editing.

## Supplementary Information

**Additional file 1: Table S1** Gene numbers in the subfamilies of ABC transporter in the genomes of *Papilio xuthus*, other 31 insects, other five arthropods and human. **Table S2**. Details of the 56 ABC transporters identified in the genome of *Papilio xuthus*. **Table S3**. Details of the Rab families identified in the genome of *Papilio xuthus* and *Bombyx mori*. **Table S4**. The target of the CRISPR-edited locus and primer for genotyping sequences. **Table S5**. Mutation rate among different individual mutants and their mutated clones across different target sites. **Table S6**. The information of RNA sequencing and data for samples. **Table S7**. The mapping information of RNA reads for all samples. **Table S8**. The mutations in the target regions from transcriptomics data. **Table S9.** Differentially expressed genes (DEGs) between the mutants of five edited genes (brown, ok, scarlet, white, lightoid) and their wild-types. **Table S10**. Summary on functions of five genes experimentally verified in this study and previously published.

**Additional file 2: Fig. S1**. No phenotypic changes were observed in wings of mutated adults of five genes induced by CRISPR/Cas9 gene editing. (A) wild type; (B) *white* mutant; (C) *scarlet* mutant; (D) *brown* mutant; (E) *ok* mutant; (F) *lightoid* mutant. Note that in the panels of B, C and E, incomplete shapes of hindwings were produced during flying; the photos of panels A, B, C, and F were taken based on live butterflies, while those of panels D and E were taken based on dried specimens. The photo credit is provided by Zhiwei Dong.

**Additional file 3: Fig. S2**. Sequence analysis for CRISPR/Cas9 mutations. (A) Knockout of three targets in three *white* mutants of fifth instar larva and of two targets in three *white* mutants of adult. (B) Knockout of two target in four *scarlet* mutants. (C) Knockout of two target in three mutants in *brown* gene. (D) Knockout of two target in three *ok* mutants. (E) Knockout of four target in three *lightoid* mutants. The above line (intron) and boxes (exon) denote gene structure and the arrow denotes transcribed direction for each gene. The regions of target sites in each exon were labeled in red. Letters in blue indicate target sequences, letters underlined indicate protospacer-adjacent motif (PAM) region, and letters in red indicate insert and substitution bases. Numbers before semicolon in brackets on the right of the sequence mean the clone number exhibiting the same mutation pattern, and numbers after the semicolon mean the length (bp) of the insertion, deletion and substitution, respectively. NA, not applicable because mutation type did not appear in the sequenced clones. WT represents wild-type.

**Additional file 4: Fig. S3**. The structure of five genes and the distribution of transcriptomic sequencing depths in their disrupted exons of mutated and wild-type individuals. (A) - (E) represent the detail information of *Px_03417_w* (*white*), *Px_03415_st* (*scarlet*), *Px_17845_w* (*brown*), *Px_17844_st* (*ok*), and *Px_17846_ltd* (*lightoid*), respectively. On the below, the structure of genes was plotted in proportion of its true length with exons in light blue blocks and target region of CRISRP in red blocks. Arrows denotes gene direction. The line chart on the top show the distribution of transcriptomic sequencing in target sites (light red and light blue blocks) and flanking regions. The light red lines and light blue lines denote mutated and wild-type individuals, respectively.

**Additional file 5: Fig. S4**. The heatmap and the principle component analysis (PCA) analysis of gene expression in mutated and wild-type individuals. (A) The heatmaps. (B) The PCA analysis.

**Additional file 6: Fig. S5**. The functional enrichment of GO term (A) and KEGG (B) pathway for all differentially expressed gene (DEGs). The different shapes represent different knocked out genes: circle, square, diamond, regular triangle, inverted triangle, indicate *Px_17845_w* (*brown*), *Px_17846_ltd* (*lightoid*), *Px_03415_st* (*scarlet*), *Px_17844_st* (*ok*), and *Px_03417_w* (*white*), respectively.

## Data Availability

All data generated or used in this study are included in this manuscript and the supplementary information files. The RNA sequencing data of mutants and wild-types used in this study have been deposited into NCBI database under BioProject Number PRJNA610787 (https://www.ncbi.nlm.nih.gov/bioproject/PRJNA610787). The genome assemblies and annotation of *Papilio xuthus* and *Bombyx mori* are available at NCBI database as BioProject ID PRJNA270384 (https://www.ncbi.nlm.nih.gov/bioproject/?term=PRJNA270384) and PRJDA20217 (https://www.ncbi.nlm.nih.gov/bioproject/PRJDA20217), respectively. The reported ABC protein sequences (Fig. 1) and Rab protein sequences (Fig. 2) of *D. melanogaster* were downloaded from FlyBase (http://flybase.org/). The reported ABC protein sequences (Fig. 1) of *B. mori* were extracted from its genome. The conserved domains of ABC protein (PF00005.24: http://pfam.xfam.org/family/PF00005.24 and PF00664.20: http://pfam.xfam.org/family/PF00664.20) and Rab protein (PF00071.19: http://pfam.xfam.org/family/PF00071.19 and PF08477.10: http://pfam.xfam.org/family/PF08477.10) are available at pfam (http://pfam.xfam.org/).

## Abbreviations

ABC: ATP-binding cassette
DEGs: Differentially expressed genes
FT: Full transporter
FPKM: Fragments per kilobase million
GO: Gene ontology
HT: Half transporter
KEGG: Kyoto encyclopedia of genes and genomes
ML: Maximum likelihood
NBDs: Nucleotide-binding domains
PCA: Principle component analysis
TMDs: Transmembrane domains
