# Supplementary figures and images for: Genome-wide identification and gene-editing of pigment transporter genes in the swallowtail butterfly Papilio xuthus
Source: BMC Genomics. 2021 Feb 17;22:120. doi: 10.1186/s12864-021-07400-z (PMC7891156; doi:10.1186/s12864-021-07400-z)

**A**

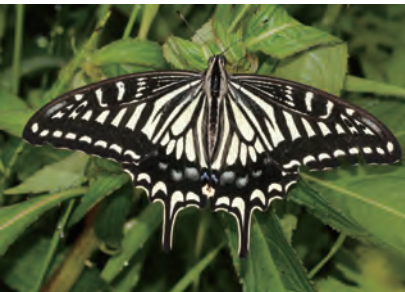

**B**

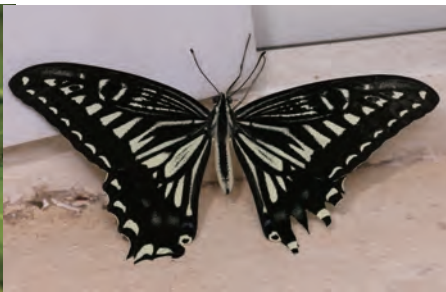

**C**

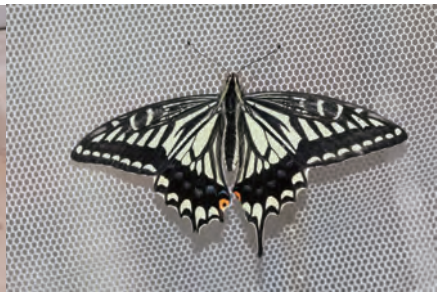

**D**

**wild type**

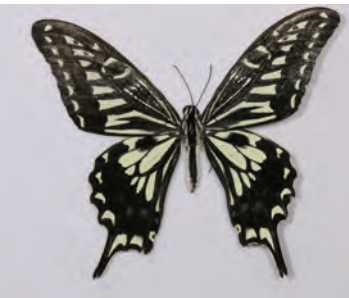

**E**

*white*

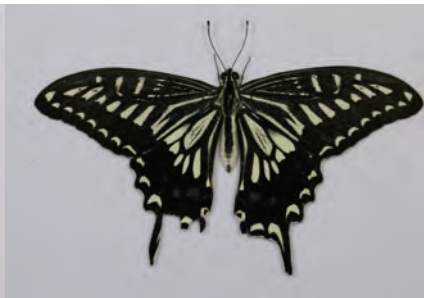

**F**

*scarlet*

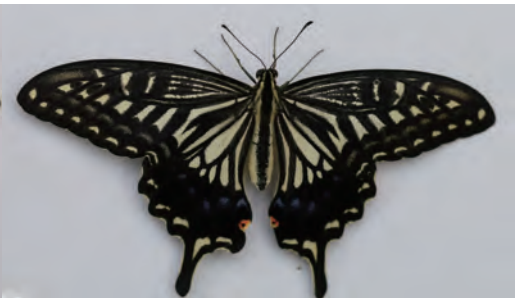

*brown*

*ok*

*lightoid*

Supplement: Supplementary file 2 — Additional file 2: Fig. S1. No phenotypic changes were observed in wings of mutated adults of five genes induced by CRISPR/Cas9 gene editing. (A) wild type; (B) white mutant; (C) scarlet mutant; (D) brown mutant; (E) ok mutant; (F) lightoid mutant. Note that in the panels of B, C and E, incomplete shapes of hindwings were produced during flying; the photos of panels A, B, C, and F were taken based on live butterflies, while those of panels D and E were taken based on dried specimens. The photo credit is provided by Zhiwei Dong. [file 12864_2021_7400_MOESM2_ESM.pdf]

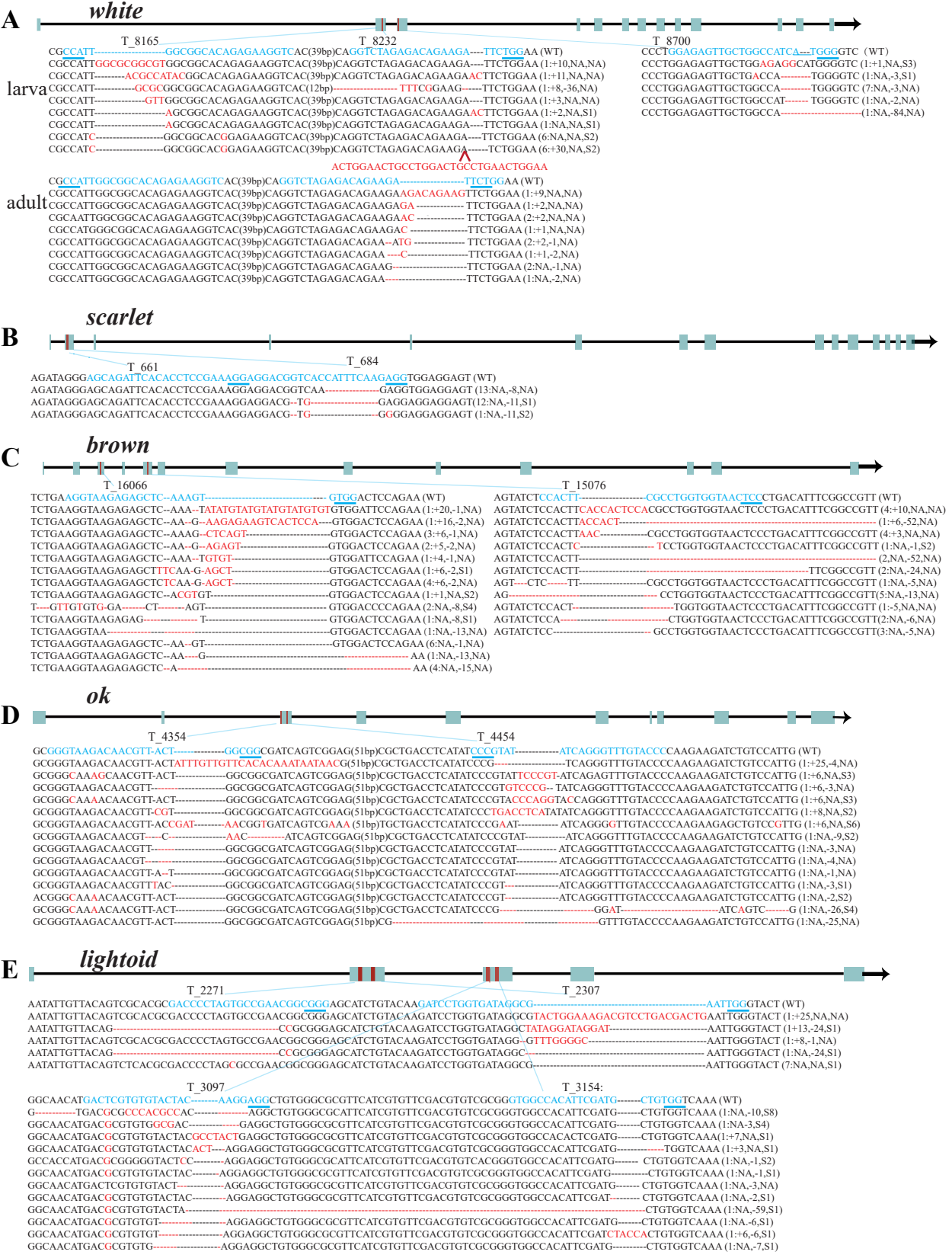

Supplement: Supplementary file 3 — Additional file 3: Fig. S2. Sequence analysis for CRISPR/Cas9 mutations. (A) Knockout of three targets in three white mutants of fifth instar larva and of two targets in three white mutants of adult. (B) Knockout of two target in four scarlet mutants. (C) Knockout of two target in three mutants in brown gene. (D) Knockout of two target in three ok mutants. (E) Knockout of four target in three lightoid mutants. The above line (intron) and boxes (exon) denote gene structure and the arrow denotes transcribed direction for each gene. The regions of target sites in each exon were labeled in red. Letters in blue indicate target sequences, letters underlined indicate protospacer-adjacent motif (PAM) region, and letters in red indicate insert and substitution bases. Numbers before semicolon in brackets on the right of the sequence mean the clone number exhibiting the same mutation pattern, and numbers after the semicolon mean the length (bp) of the insertion, deletion and substitution, respectively. NA, not applicable because mutation type did not appear in the sequenced clones. WT represents wild-type. [file 12864_2021_7400_MOESM3_ESM.pdf]

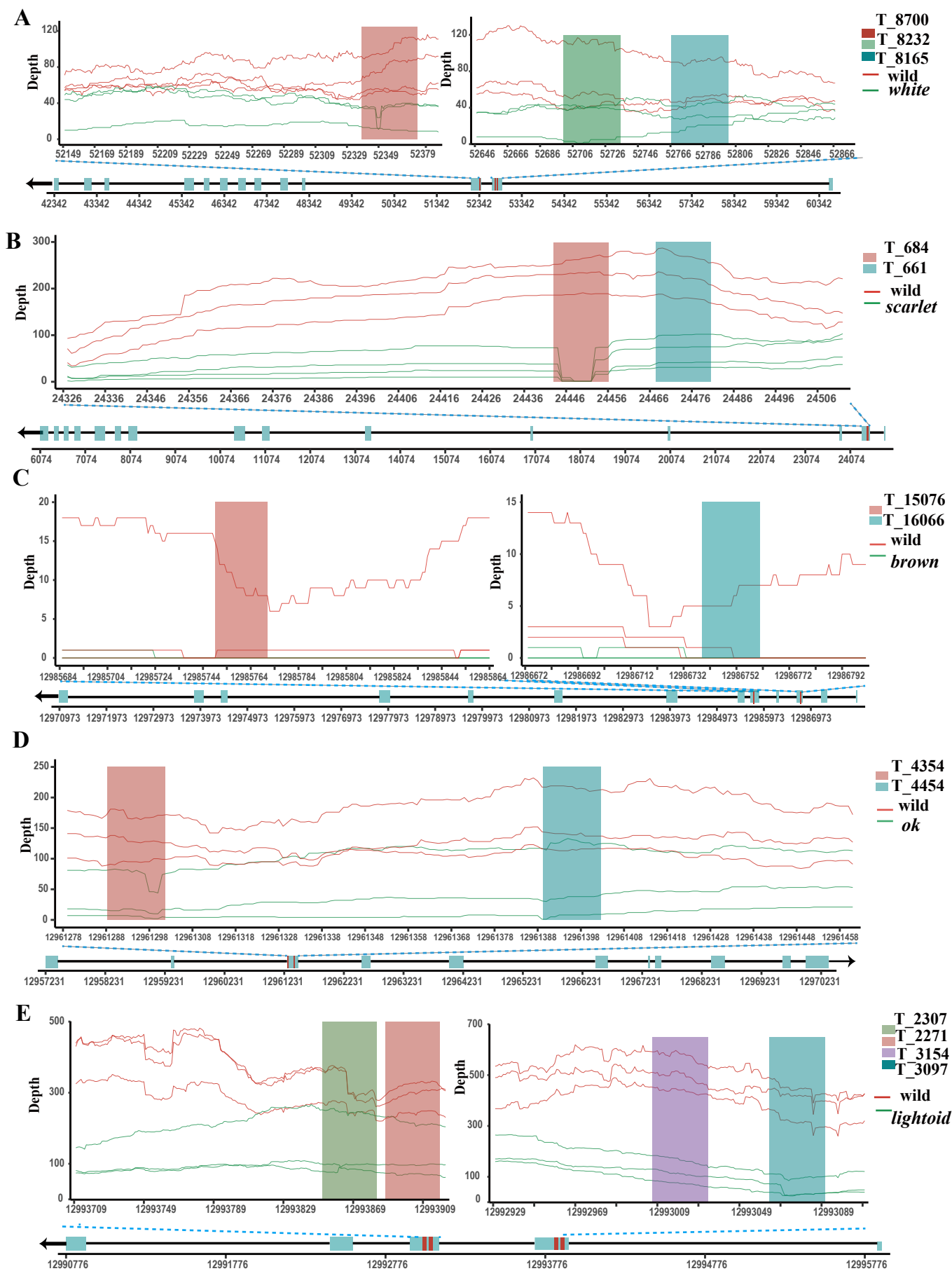

Supplement: Supplementary file 4 — Additional file 4: Fig. S3. The structure of five genes and the distribution of transcriptomic sequencing depths in their disrupted exons of mutated and wild-type individuals. (A) - (E) represent the detail information of Px_03417_w (white), Px_03415_st (scarlet), Px_17845_w (brown), Px_17844_st (ok), and Px_17846_ltd (lightoid), respectively. On the below, the structure of genes was plotted in proportion of its true length with exons in light blue blocks and target region of CRISRP in red blocks. Arrows denotes gene direction. The line chart on the top show the distribution of transcriptomic sequencing in target sites (light red and light blue blocks) and flanking regions. The light red lines and light blue lines denote mutated and wild-type individuals, respectively. [file 12864_2021_7400_MOESM4_ESM.pdf]

A

*white*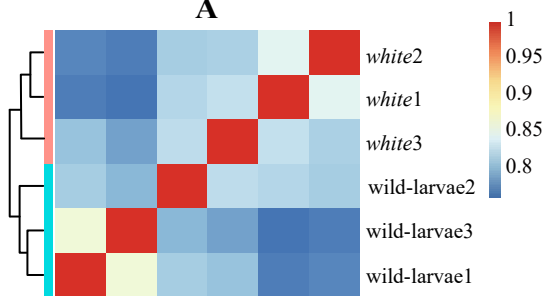

B

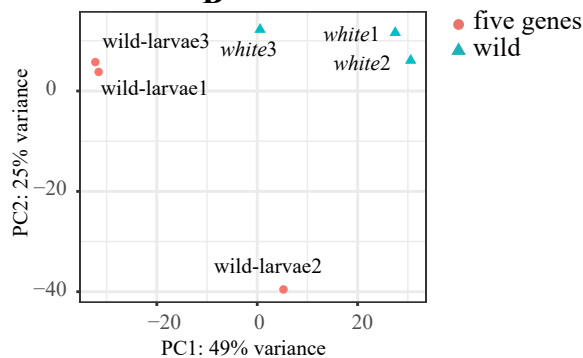*scarlet*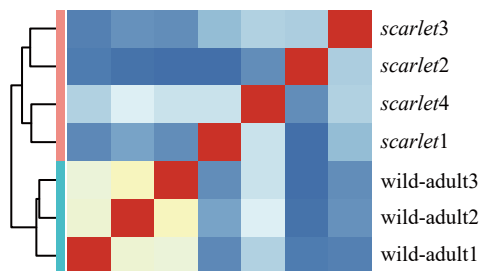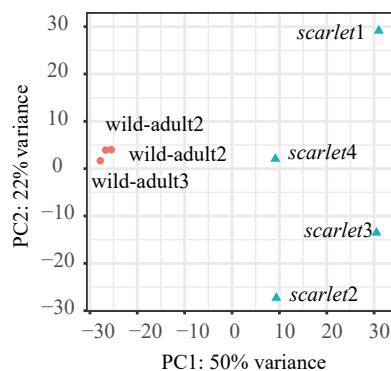*brown*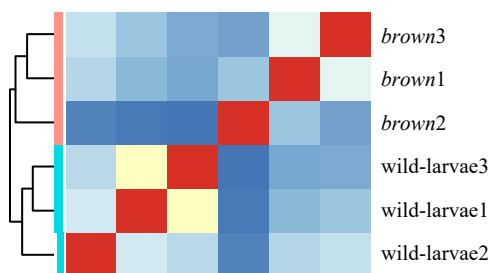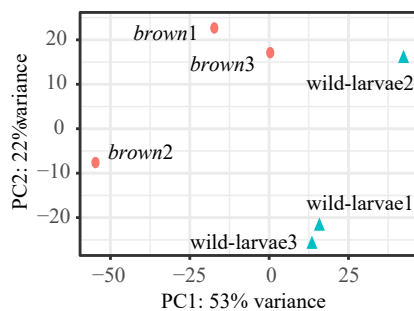*ok*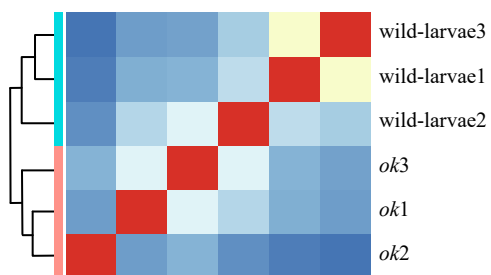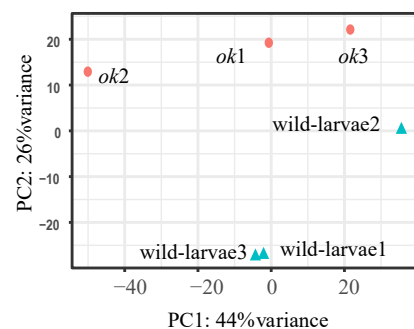*lightoid*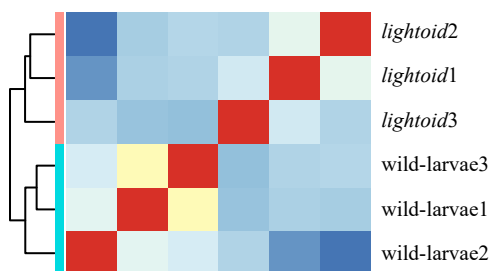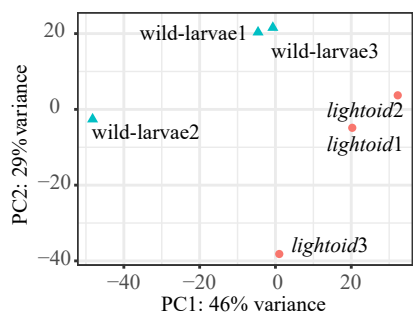

Supplement: Supplementary file 5 — Additional file 5: Fig. S4. The heatmap and the principle component analysis (PCA) analysis of gene expression in mutated and wild-type individuals. (A) The heatmaps. (B) The PCA analysis. [file 12864_2021_7400_MOESM5_ESM.pdf]

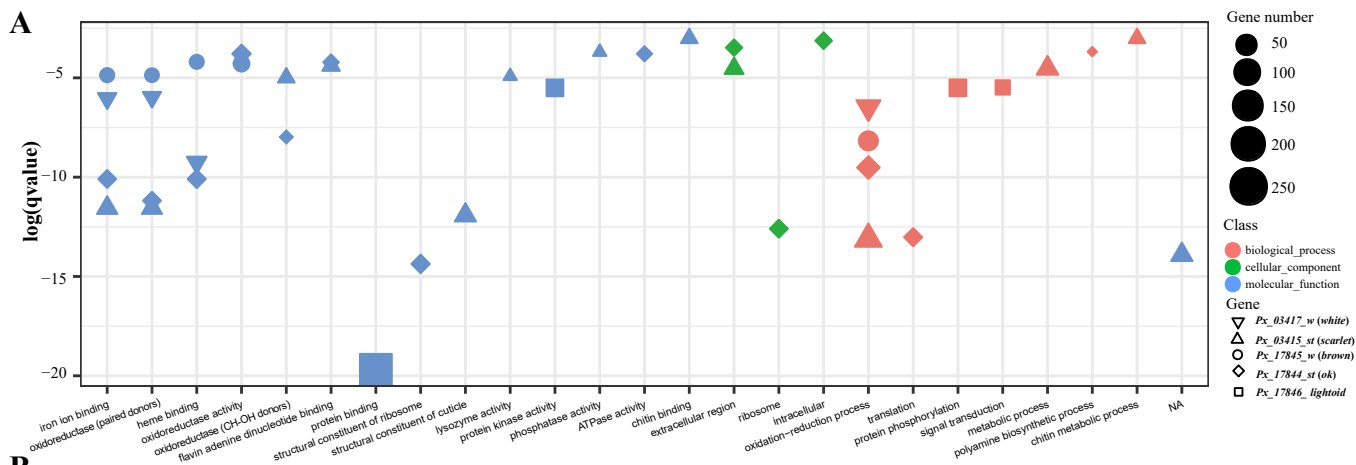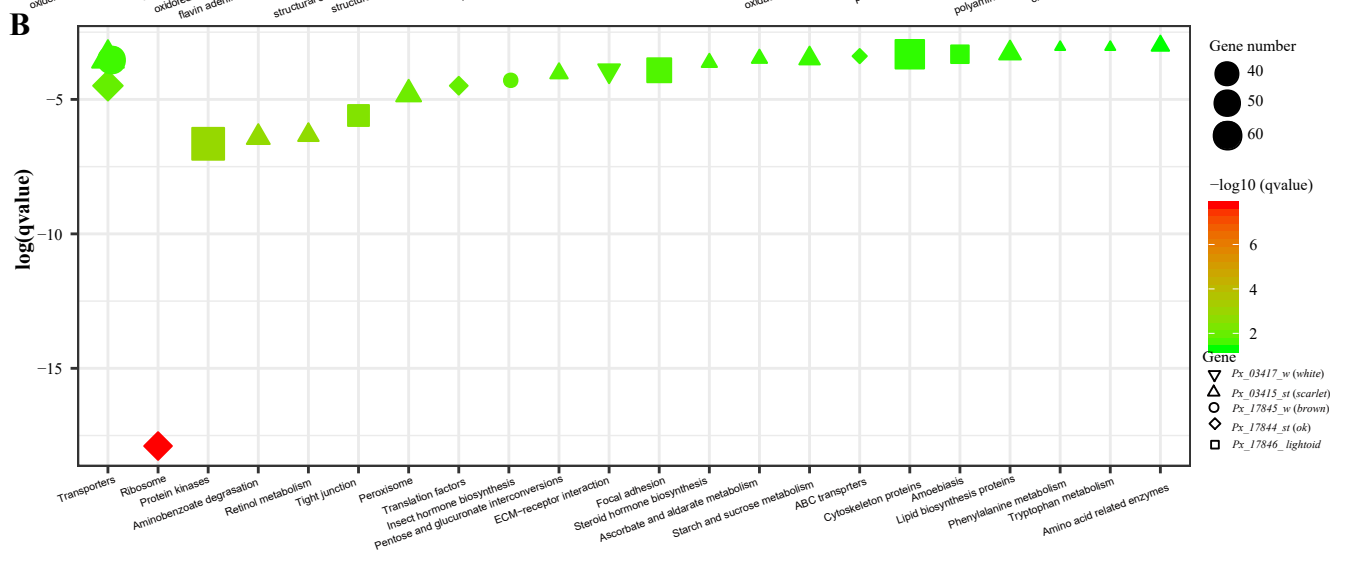

Supplement: Supplementary file 6 — Additional file 6: Fig. S5. The functional enrichment of GO term (A) and KEGG (B) pathway for all differentially expressed gene (DEGs). The different shapes represent different knocked out genes: circle, square, diamond, regular triangle, inverted triangle, indicate Px_17845_w (brown), Px_17846_ltd (lightoid), Px_03415_st (scarlet), Px_17844_st (ok), and Px_03417_w (white), respectively. [file 12864_2021_7400_MOESM6_ESM.pdf]
